# Supplementary material for: Early Reoperation Rates and Its Risk Factors after Instrumented Spinal Fusion Surgery for Degenerative Spinal Disease: A Nationwide Cohort Study of 65,355 Patients
Source: J Clin Med. 2022 Jun 10;11(12):3338. doi: 10.3390/jcm11123338 (PMC9225055; doi:10.3390/jcm11123338)
Supplement: Supplementary file 1 [file jcm-11-03338-s001.zip › jcm-1726373-supplementary.pdf]

Table S1. ICD-10 codes for comorbidities including Charlson comorbidities index items and scores.

Type of comorbidities	Category	ICD-10 codes	Scores
Included in Charlson comorbidity index	Myocardial infarction	I21, I22, I25.2	1
	Congestive heart failure	I09.9, I11.0, I13.0, I13.2, I25.5, I42.0, I42.5-I42.9, I43, I50, P29.0	1
	Peripheral vascular disease	I70, I71, I73.1, I73.8, I73.9, I77.1, I79.0, I79.2, K55.1, K55.8, K55.9, Z95.8, Z95.9	1
	Cerebrovascular disease	G45, G46, I60-I69, H34.0	1
	Dementia	F00-F03, G30, F05.1, G31.1	1
	Chronic pulmonary disease	I27.8, I27.9, J40-J47, J60-J67, J68.4, J70.1, J70.3	1
	Rheumatologic disease	M05, M06, M31.5, M32-M34, M35.1, M35.3, M36.0	1
	Peptic ulcer	K25-K28	1
	Hemiplegia or paraplegia	G04.1, G11.4, G80.1, G80.2, G81, G82, G83.0, G83.1,<br>G83.2, G83.3, G83.4, G83.9	2
	Diabetes without complication	E10.0, E10.1, E10.6, E10.8	1
	Diabetes with complication	E10.9, E11.0, E11.1, E11.6, E11.8, E11.9, E12.0, E12.1,<br>E12.6, E12.8, E12.9, E13.0 E13.1, E13.6, E13.8, E13.9,<br>E14.0, E14.1, E14.6, E14.8, E14.9	2
	Mild liver disease	B18, K70.0-K70.3, K70.9, K71.3-K71.5, K71.7, K73, K74, K76.0, K76.2-K76.4, K76.8, K76.9, Z94.4	1
	Moderate to severe liver disease	I85.0, I85.9, I86.4, I98.2, K70.4, K71.1, K72.1, K72.9,<br>K76.5-K76.7	3
	Moderate to severe renal disease	I12.0, I13.1, N03.2-N03.7, N05.2-N05.7, N18, N19, N25.0, Z49.0-Z49.2, Z94.0, Z99.2	2
	Any malignancy<br>(including leukemia or lymphoma)	C00-C26, C30-C34, C37-C41, C43, C45-C58, C60-C76<br>C81-C85, C88, C90-C97	2
	Metastatic solid tumor	C77-C80	6
	Acquired immunodeficiency syndrome	B20-B22, B24	6
Others	Osteoporosis	M80-M82	-
	Parkinson disease	G20	-
	End stage renal disease	E10.22, E11.22, E12.22, E13.32, E14.22, N18.5, Z99.2<br>(V001, V003)	-

Table S2. HIRA therapeutic codes for transfusion.

Type of transfusion	HIRA therapeutic code
Autologous transfusion	X6001-X6008
Allogenous transfusion	X2021, X2022, X2031, X2032, X2091, X2092, X2111, X2131, X2132, X2512, X2515, X9006-9007

Table S3. HIRA general name codes for used steroids.

Category of steroid	Type of steroid	ATC code	HIRA general name code
Oral steroid	deflazacort 6 mg	H02AB13	140801ATB
	dexamethasone 0.5 mg	H02AB02	141901ATB
	dexamethasone 0.75 mg	H02AB02	141903ATB
	betamethasone 0.25 mg + d-chlorpheniramine 2 mg	H02AB01	296900ATB
	hydrocortisone 10 mg	H02AB09	116401ATB
	hydrocortisone 5 mg	H02AB09	170901ATB
	methylprednisolone 4 mg	H02AB04	193302ATB
	methylprednisolone 1 mg	H02AB04	193305ATB
	prednisolone 5 mg	H02AB06	217001ATB
	triamcinolone 1 mg	H02AB08	243201ATB
	triamcinolone 2 mg	H02AB08	243202ATB
	triamcinolone 4 mg	H02AB08	243203ATB
	fludrocortisone 100 µg	H02AA02	160201ATB
Intravenous steroid	dexamethasone 4 mg	H02AB02	142030BIJ
	dexamethasone 4 mg	H02AB02	142230BIJ
	dexamethasone 5 mg	H02AB02	142232BIJ
	betamethasone 4 mg	H02AB01	116530BIJ
	hydrocortisone 100 mg	H02AB09	171201BIJ
	methylprednisolone 125 mg	H02AB04	193601BIJ
	methylprednisolone 40 mg	H02AB04	193603BIJ
	methylprednisolone 500 mg	H02AB04	193604BIJ
	triamcinolone 10 mg	H02AB08	243336BIJ
	triamcinolone 40 mg	H02AB08	243335BIJ
	triamcinolone 40 mg	H02AB08	243337BIJ

Table S4. Risk factors for reoperation within one year after instrumented spinal fusion surgery for degenerative spinal disease: multivariable analysis.

Variables	Categories	Model 1		Model 2 (backward)		Model 3 (bootstrap adjusted)	Bias (%)
		Adjusted odds ratio (95% confidence interval)	p-value	Adjusted odds ratio (95% confidence interval)	p-value	Adjusted odds ratio (95% confidence interval)	
Age	50-69 vs 20-49 years	1.30 (1.05 - 1.61)	0.018	1.31 (1.06 - 1.63)	0.014	1.31 (1.10 - 1.57)	-0.3%
	70-79 vs 20-49 years	1.51 (1.19 - 1.92)	<0.001	1.54 (1.21 - 1.95)	<0.001	1.56 (1.27 - 1.92)	1.1%
	80+ vs 20-49 years	1.46 (1.00 - 2.13)	0.053	1.49 (1.02 - 2.18)	0.039	1.46 (1.06 - 2.01)	2.4%
Sex	Male vs female	1.79 (1.58 - 2.03)	<0.001	1.79 (1.58 - 2.03)	<0.001	1.33 (1.26 - 1.40)	25.6%
Region	Rural vs urban	0.97 (0.83 - 1.15)	0.750				
Hospital	General vs tertiary	1.40 (1.19 - 1.65)	<0.001	1.39 (1.18 - 1.63)	<0.001	1.39 (1.22 - 1.62)	1.3%
	Others vs tertiary	1.08 (0.92 - 1.26)	0.360				
Charlson comorbidity index score	3-5 vs 0-2	1.01 (0.75 - 1.34)	0.972				
	≥6 vs 0-2	0.81 (0.48 - 1.39)	0.450				
Comorbidities	Congestive heart failure	1.47 (1.12 - 1.93)	0.005	1.46 (1.12 - 1.90)	0.005	1.22 (1.08 - 1.36)	48.0%
	Cerebrovascular disease	1.01 (0.82 - 1.24)	0.603				
	Chronic pulmonary disease	1.08 (0.80 - 1.46)	0.603				
	Rheumatologic disease	1.36 (1.06 - 1.74)	0.015	1.35 (1.06 - 1.72)	0.016	1.17 (1.05 - 1.27)	47.4%
	Peptic ulcer disease	1.26 (1.08 - 1.46)	0.003	1.24 (1.08 - 1.44)	0.003	1.11 (1.04 - 1.18)	49.8%
	Diabetes, uncomplicated	1.21 (1.04 - 1.40)	0.016	1.20 (1.04 - 1.38)	0.013	1.19 (1.08 - 1.31)	6.0%
	Diabetes, complicated	1.43 (1.08 - 1.87)	0.011	1.39 (1.13 - 1.70)	0.002	1.09 (1.02 - 1.16)	72.8%
	Renal disease	1.48 (1.00 - 2.21)	0.056				
	Parkinson disease	2.04 (1.29 - 2.22)	0.002	2.03 (1.29 - 2.20)	0.002	1.43 (1.12 - 1.80)	49.9%
	End stage renal disease	2.19 (1.20 - 4.01)	0.011	3.03 (1.85 - 4.98)	<0.001	1.85 (1.35 - 2.90)	44.5%
Allogenous transfusion		1.47 (1.28 - 1.69)	<0.001	1.47 (1.28 - 1.69)	<0.001	1.21 (1.15 - 1.28)	17.6%
Systemic steroid	within 2 weeks vs none	1.43 (1.26 - 1.62)	<0.001				
	Over 2 weeks vs none	1.60 (0.63 - 4.03)	0.323	1.43 (1.26 - 1.62)	<0.001	1.43 (1.29 - 1.56)	0.3%
Surgical approach	Cervical anterior vs lumbar posterior	0.69 (0.56 - 0.86)	0.001	0.69 (0.56 - 0.86)	0.001	0.70 (0.58 - 0.83)	-1.5%
	Cervical posterior vs lumbar posterior	0.42 (0.24 - 0.74)	0.003	0.42 (0.24 - 0.74)	0.003	0.40 (0.21 - 0.62)	5.6%
	Thoracic anterior vs lumbar posterior	4.29 (1.29 - 14.28)	0.018	4.35 (1.31 - 14.46)	0.005	0.15 (0.00 - 7.46)	96.5%

Cage	1.27 (1.09 - 1.48)	0.002	1.27 (1.09 - 1.47)	0.002	1.12 (1.04 - 1.21)	11.8%

All significant independent variables ($p<0.05$) from the univariable analysis were included in this multivariable model. Relative bias was estimated as the difference between the mean bootstrapped regression coefficient estimates (model 3) and the mean parameter estimates of multivariable model (model 2) divided by the mean parameter estimates of multivariable model (model 2).
